# Design of a Digital-Based, Multicomponent Nutrition Guidance System for Prevention of Early Childhood Obesity

**DOI:** 10.1155/2016/5067421

**Published:** 2016-08-18

**Authors:** Keriann H. Uesugi, Anne M. Dattilo, Maureen M. Black, Jose M. Saavedra

**Affiliations:** ^1^Nestlé Nutrition, 12 Vreeland Road, Florham Park, NJ 07932, USA; ^2^Department of Pediatrics, University of Maryland School of Medicine, 737 W Lombard Street No. 161, Baltimore, MD 21201, USA; ^3^RTI International, East Cornwallis Road, P.O. Box 12194, Research Triangle Park, NC 27709-2194, USA; ^4^Nestlé S.A., Avenue Nestlé 55, 1800 Vevey, Switzerland

## Abstract

Interventions targeting parenting focused modifiable factors to prevent obesity and promote healthy growth in the first 1000 days of life are needed. Scale-up of interventions to global populations is necessary to reverse trends in weight status among infants and toddlers, and large scale dissemination will require understanding of effective strategies. Utilizing nutrition education theories, this paper describes the design of a digital-based nutrition guidance system targeted to first-time mothers to prevent obesity during the first two years. The multicomponent system consists of scientifically substantiated content, tools, and telephone-based professional support delivered in an anticipatory and sequential manner via the internet, email, and text messages, focusing on educational modules addressing the modifiable factors associated with childhood obesity. Digital delivery formats leverage consumer media trends and provide the opportunity for scale-up, unavailable to previous interventions reliant on resource heavy clinic and home-based counseling. Designed initially for use in the United States, this system's core features are applicable to all contexts and constitute an approach fostering healthy growth, not just obesity prevention. The multicomponent features, combined with a global concern for optimal growth and positive trends in mobile internet use, represent this system's future potential to affect change in nutrition practice in developing countries.

## 1. Introduction

Problems in nutrition and growth in early life have increasingly been implicated in long term health outcomes with devastating consequences to human capital. Undernutrition's effects in early life are well documented and culminate in increased growth faltering, morbidity, and mortality as well as impairment in cognitive development and diminished work capacity in adulthood [1, 2]. These negative effects are best prevented in the first 1000 days of life, as attempts to treat after that time are less effective [3].

A parallel scenario is surfacing related to overweight and obesity in early life. Current research supports consistent associations of maternal weight gain during gestation, large for gestational age birth size, and rapid weight gain during infancy with BMI, adiposity, or risk of overweight/obesity in childhood through adulthood [4–6]. Consequences of rapid weight gain in infancy are also linked to adverse cardiovascular and metabolic outcomes later in life [7–10]. At present, efforts to prevent and treat obesity during childhood have mixed success [11, 12]. Thus, prevention of both over and undernutrition is a global concern, with interventions beginning during the first 1000 days of life.

Results from intervention trials for prevention of early childhood obesity within the first two years of life are limited [11, 13–15], and few begin during gestation. Although three large randomized controlled trials (RCT) [16–19] enrolled new mothers to educational interventions with the goal of early obesity prevention, results ranged from a significant 0.29-unit decrease in BMI at 2 years [16] and lower BMI *z*-scores plus decreased odds of rapid weight gain at 13.7 months but not at 2 years [18, 19] to no significant difference in BMI *z*-score at 20 months [17]. Even for the trials that found significant effects at 2 years, those effects diminished by 5 years with no further intervention [20, 21]. Following these first efforts with mixed results and to potentially achieve a greater effect size, theoretically driven interventions that increase the likelihood of sustainability are needed.

High rates of overweight also urge a focus on developing scalable interventions that reach children prior to the onset of either insufficient or excess weight gain [11]. Globally, 6.1% of children aged 0–5 years are overweight or obese (proportion of children with weight-for-height above 2 standard deviations) [22], and projections indicate an increase to 9.9% in 2025 [2]. In the US, 7.1% of children aged 0–2 years have weight-for-length >95th percentile and the prevalence of overweight or obesity is 22.8% among children aged 2–5 years [23]. Therefore, there is need for innovative and effective interventions with delivery mechanisms that can be disseminated to the wider population.

Digital-based interventions are increasingly feasible for such scale-up efforts. These web and mobile-based programs help to avoid the limitations of prior interpersonal interventions which often affect coverage, dose, and fidelity [24–29]. Parents, especially mothers, spend a significant amount of time on the internet and frequently use it to seek parenting and child health related information [30–32]. Thus, the target population would be familiar with such a format to deliver this type of intervention.

The objective of this paper is to provide a detailed description of a digital-based, multicomponent nutrition guidance system designed to improve feeding and related practices by first-time mothers for prevention of obesity and promotion of optimal growth in their children during the first two years of life. This digital-based system is designed to be scaled up to reach all populations in need and be easily adapted to accommodate varying sociocultural contexts for the prevention of over- and undernutrition during the first 1000 days.

## 2. Methods

Development of the multicomponent nutrition guidance system for healthy growth and prevention of early childhood obesity was informed by the World Health Organization's (WHO) health education theoretical concepts and strategies [33] and guided by Contento's Procedural Model for Nutrition Education [34] involving four process components: (1) identification of modifiable factors which could be target behaviors; (2) identification of potential mediators; (3) selection and justification of theoretical model; (4) and design of the intervention. We further sought to explore generalizability of the nutrition guidance system and a digital delivery format. An overarching goal for our process was to follow a rigorous method to substantiate all components of the final intervention, from development of educational content to intervention delivery.

Systematic literature reviews were conducted for components 1 and 2. Methods for the literature review to identify target behaviors (i.e., modifiable factors) were previously described by Dattilo et al. [35]. For the identification of potential mediators, inclusion criteria included (1) studies published in English language from January 1, 2000, through September 30, 2012; (2) studies conducted in high income countries; (3) mean age of children in the sample less than 5 years; (4) maternal age ≥ 18 years; and (5) sample size > 10. Literature included peer-reviewed articles, public policy statements, and publically available guidance from the WHO, American Academy of Pediatrics (AAP), and the US government departments of Health and Human Services and Agriculture (DHHS and USDA, resp.).

## 3. Results and Discussion

### 3.1. Component 1: Identification of Target Behaviors

 The modifiable factors that influence healthy growth of infants defined as dietary, feeding, and care practices and could be addressed in interventions beginning at birth have been previously identified by our group [35]. These modifiable factors formed the basis for the current intervention's target behaviors and were assigned to eight core messages of the nutrition guidance system: (1) provide breastmilk; (2) utilize responsive feeding practices; (3) provide nutritious complementary foods and beverages at the appropriate developmental stage; (4) exclude sugar sweetened beverages; (5) foster healthy eating behaviors through shared family meals and mealtime routines; (6) limit TV and screen viewing time; (7) provide opportunities for physical activity; and (8) ensure that the infant/toddler has adequate sleep.

### 3.2. Component 2: Identification of Potential Mediators

Potential mediators, defined as underlying determinants that precede behaviors, were identified via systematic literature reviews for each of the aforementioned core messages. The resultant potential mediators and cognitive variables included knowledge, attitudes, beliefs, self-efficacy, social norms, and skills, as well as environmental constraints that influenced whether or not a target behavior was performed. Research findings related to the mediators were summarized onto Research-Based Content Tables for each core message. Findings were summarized and categorized into four types of mediators: knowledge; instruction; facilitator; or barrier.

### 3.3. Component 3: Selection and Justification of Theoretical Model

Nutrition education interventions use theories from the ecological, social, and psychological sciences to help identify the constructs or mediating variables that influence the behavior of interest [36] and then apply theoretically specified techniques to modify the mediating variables resulting in behavioral adoption [37–39]. Social Cognitive Theory (SCT), Theory of Planned Behavior (TPB), and the Health Belief Model (HBM) were selected for the multicomponent feeding guidance program based on their application in observational and qualitative studies [40–51] and successful implementation within intervention trials [17, 19, 52–55] related to infant, toddler, and preschool-aged feeding behaviors, as well as their strengths in promoting motivation to perform behavior (Theory of Planned Behavior and Health Belief Model) and supporting capacity to act on the behavior (Social Cognitive Theory) [56]. SCT has been successfully incorporated within interventions to prevent early introduction of solids [31], increase use of positive responsive feeding practices, decrease consumption of sweet snacks, and lower daily television viewing time [17, 19] and is being utilized in upcoming interventions targeted at the early prevention of child obesity [57–60]. The self-efficacy construct from SCT has been found to be predictive of breastfeeding intention and duration [40–43], providing nutritious complementary foods and beverages [44], decreasing sweetened beverage consumption [44], and promoting physical activity while limiting screen time [44].

Constructs from TPB were frequently cited to explain motivation and intention related to the core messages. Constructs such as attitudes, perceived behavioral control, moral norms, and subjective norms have been linked with providing breastmilk [45, 48, 49], provision of nutritious complementary foods at appropriate developmental stage [46, 48, 50], and foster healthy eating through shared family meals and mealtime routines [47]. Thus, those constructs were maintained in the overall theoretical model related to behavioral intention.

The Health Belief Model was included in addition to TPB to increase behavioral intention due to its theoretical framework to understand barriers and facilitators to family meals [51] and its unique constructs of perceived susceptibility and perceived severity [56]. The perceived risk of early childhood obesity is a central component because of prevailing beliefs about larger infants being healthier or better [48, 61, 62], tendencies to practice unresponsive feeding such as pressuring, despite acknowledging infant satiety cues [63, 64], failure to perceive overweight children as overweight [62, 65], and a general belief that young children will grow out of any early overweight or obese status [61, 66].

**Figure 1:**
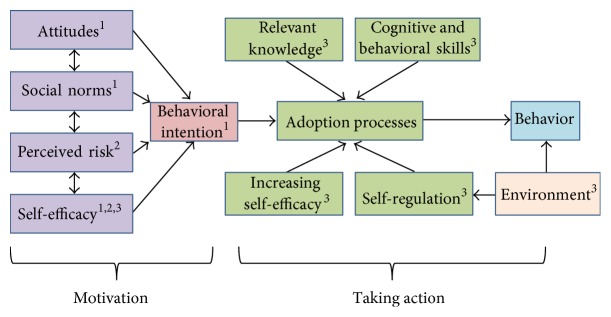
Theoretical model for the nutrition guidance system based on (1) Theory of Planned Behavior, (2) Health Belief Model, and (3) Social Cognitive Theory.

From the Research-Based Content Tables, each research finding and its classification as knowledge, instruction, facilitator, or barrier were linked to a construct within SCT, TPB, and HBT (Table 1). A detailed theoretical framework for this digital-based nutrition guidance system is shown in Figure 1.

Two additional frameworks, anticipatory guidance and motivational interviewing, were also incorporated. Anticipatory guidance was selected to inform the timing of messages and content delivery. Since anticipatory guidance is the prevailing framework for pediatric well-visits during childhood in the US [67–69] and previously incorporated within other interventions testing early obesity prevention [17, 19], this approach was included in the multicomponent nutrition guidance system as a method to proactively deliver components of core messages to parents during the period just prior to when the issue will be developmentally relevant to the infant or child. Similarly, motivational interviewing is a client centered communication technique that has been recommended and found effective for treatment of pediatric obesity in clinical settings [70, 71] and fits within the standards of practice for registered dietician nutritionists (RDNs) [72].

### 3.4. Component 4: Design of Intervention

**Figure 2:**
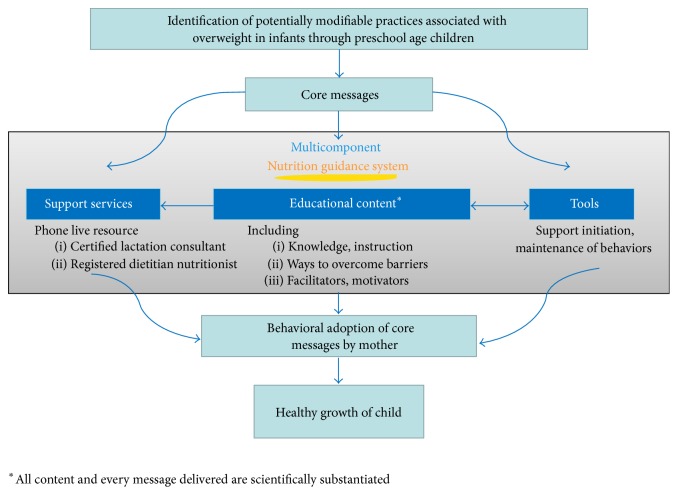
Conceptual framework for the nutrition guidance system.

The multicomponent nutrition guidance system includes digitally based educational content and tools, plus telephone-based professional support from certified lactation consultants (CLC) and registered dietitian nutritionists (RDNs) as depicted in the system conceptual framework (Figure 2). Educational content is intended to provide the required knowledge and instruction as well as address the barriers and facilitators associated with implementing the core messages. The tools are meant to help mothers initiate and maintain behaviors within the core messages. Lastly, the telephone-based professional support is available to help mothers with remaining needs related to adopting the core messages and reinforce content delivered digitally using a motivational interviewing approach. Mothers initiate contact with the CLC or RDN by calling a toll-free number or scheduling an appointment on the website for the CLC or RDN to call them at a certain time. These components are consistent with the theoretical frameworks, and both the educational content and tools are designed to address the underlying theoretical constructs to affect behavioral adoption. The following sections describe the considerations undertaken with regard to development of content and tools, timing of delivery, delivery format, and generalizability to diverse audiences.

### 3.5. Development of Content and Tools

The intervention content team, composed of Pediatricians, two Ph.D. in nutrition, and Registered Dietitian Nutritionists with expertise in nutrition education and maternal and childhood nutrition formulated the content and delivery timeline for the digital-based intervention. Additional input was incorporated from an academic advisory board, consumer communications professionals, and a creative agency group regarding aesthetic layout and digital framework.

The content and tools include articles, emails, videos, quizzes, infographics, printable and interactive trackers, and a goal setting tool and were developed to constitute the theoretically specified techniques to address the constructs of SCT, TPB, and HBM and influence behavioral adoption (Table 2) [37, 56, 73].

Utilizing a rigorous process to develop the written copy and ensure thorough substantiation of all included content, the first step within content development was to complete the Research-Based Content Tables for each of the 8 core messages which included research findings related to underlying behavioral determinants of each core message. Content was divided into 13 different modules to be regularly delivered over the course of the first two years, with the first module delivered prenatally. The second step was to prioritize up to 10 messages per core message from the content tables as priority messages to be emphasized within each of the modules. Each message was scheduled for when it would be presented to mothers as a preview in a module, when it would be discussed at length, and when it would be reviewed in later modules. This schedule or cadence of messages allowed for quick review to ensure appropriate timing and adequacy of emphasis within the intervention. The last step was creation and approval by the intervention team of final copy decks including headlines, subheadings, full copy, and photo image files to directly populate the website pages. Consideration was made with regard to scientific accuracy, adherence to nutrition education constructs, appropriate reading level (average no greater than 8th grade) [74, 75], and tone.

Emails, developed by the intervention team, were scheduled to be sent to notify mothers when the next module was available for viewing and to periodically remind mothers of assets in the current learning module to encourage them to visit the website. Text messages are also sent to mothers who have opted to receive them, and the texts reinforce messages from the current educational module and encourage mothers to visit the website. Emails and text messages are programmed to be automatically sent from the system based on the infant's birthdate and other pieces of personal information from the dyad's profile stored in the website database.

The tools were developed to increase behavioral capacity and self-regulation, constructs from SCT affecting the ability to take action and maintain newly adopted behaviors [56]. The interactive digital tools include an interactive growth tracker, a goal setting tool, and a menu planner. The growth tracker supports self-monitoring or in this case “maternal-monitoring” and allows mothers to input their child's weight and length at any point, and the tracker will plot the child's weight-for-length percentile. If a child's weight-for-length percentile is outside of the 5th–85th percentile for healthy growth and/or has crossed two percentiles, the mother will see a pop-up box with copy suggesting that she consult her child's health care provider. Accuracy of measurements is not ensured, but instructions on the growth tracker will include suggestions to use measurements taken at health care provider visits so this tool can be used to review a child's progress over time at home. The goal setting tool is designed to assist mothers in achieving self-directed larger goals via performance of smaller tasks [76]. A menu planner allows for the mother to plan meals for the week by accessing a list of nutritious meal and snack options and adding them to different days of the week. The menu planner can change from infant only use to a family meal planner as the infant transitions to family foods as a way to prompt parents to model healthy eating for their child during family meals and to ensure continued provision of nutrient dense meals and snacks to the child [77]. Noninteractive, printable tools are available as well including a breastfeeding tracker, diaper tracker, and taste tracker (to track number of exposures to new foods and baby's reactions).

### 3.6. Sequence of Educational Content Delivery

Based on the anticipatory guidance framework [67], a sequential and anticipatory timing of delivery was incorporated. The educational content and tools are delivered approximately every two months for a total of 13 intervention modules known as “Building Blocks.” Building Blocks are delivered during the third trimester of pregnancy, soon after birth, and every 2 months thereafter until the child is 22 months of age. At enrollment in the intervention, future Building Blocks are locked from viewing until the child reaches the appropriate age-based stage in order to focus maternal attention on the necessary content for the child's current age (Table 3). The option for consultation with RDN and/or CLC is encouraged throughout the intervention.

### 3.7. Delivery Format

The nutrition guidance system utilizes only digital-based vehicles for delivering the educational content and tools. A website is the repository of all the content and tools and houses an online-scheduling system for making appointments with the professional support team. The website is optimized for use on a mobile device.

Previously, only person-to-person formats (including clinic-based, group education-based, and home-based) have been utilized in interventions to promote healthy growth and appropriate dietary, feeding, screen time, and sleep behaviors among the 0–2-year-old population. While studies have had some success in affecting weight status [13, 16, 18, 78] and behaviors [14–18, 69, 79], these person-to-person delivery formats have limitations which make national and global scale-up time consuming, resource draining, and potentially less effective. Clinic-based interventions can be limited by lack of provider time [24, 80–82], inadequate provider training in nutrition counseling [24–26], and beliefs that public health education is not part of the provider's scope of work [24, 25, 27]. Group education settings have been plagued by low attendance either due to lack of transportation and childcare [28, 29] or inability to coordinate schedules around work or school [19, 29]. Finally, while home-based interventions, usually consisting of a trained nurse visiting the family home, are the most intensive intervention delivery method, they also require the most resources including hiring, training, and supervising a large staff plus travel costs for individual home visits. Thus, scale-up would entail a high cost in order to overcome these limitations and achieve sufficient coverage, dose, and fidelity needed to maintain effectiveness of the intervention.

Digital-based interventions are likely to be an effective and acceptable alternative for the target population. Digital-based interventions have been effective for behavior modification in adult populations including interventions targeting diet, physical activity, and weight [83–86]. Utilization of theory and behavior change techniques also enhances their effectiveness [73, 86, 87]. Although several digital-based interventions have targeted this population of parents and child, only one has explicitly focused on prevention of overweight in 0–2-year-old children. Denney-Wilson et al. are currently testing a website and smartphone app-based intervention to improve infant feeding and parenting behaviors among socioeconomically disadvantaged parents of infants aged 0–9 months in Australia [88]. Interventions have addressed breastfeeding [89, 90], newborn care [90, 91], parenting [90], maternal fruit and vegetable consumption [92], and toddler safety [93]. Digital-based education is also used in national maternal and child health programs such as https://www.wichealth.org/ for the Supplemental Nutrition Program for Women, Infants, and Children (WIC) [94] and the Text4Baby program [95] in the United States and the Hello World email-based program in Netherlands [96, 97].

General media trends indicate rapid, consolidated movement towards use of digital over print. As of January 2014, ninety-seven percent of adults in the US aged 18–29 and 93% of adults aged 30–49 were internet users [98]. Eighty-five percent of 18–29-year-old adults own smartphones, and more African Americans and Hispanic Americans own smartphones than White Americans demonstrating that this trend reaches multiple sectors of the population [99]. Internet was the only media (including magazines, newspapers, TV, radio, internet, email, and cell phone) which women increased their use of after becoming mothers [31]. Additionally, mothers spend twice as much time online as the general population [31].

Research also points to greater parenting-related and health information seeking online further indicating likely acceptance of the digital format. More than half of mothers surveyed sought parenting-related information from digital sources [31], and 76% said that they look up parenting advice monthly or more often on their mobile device or tablet [30]. Previous studies have found that younger mothers, especially first-time mothers, are more likely than older and experienced mothers to use and trust the internet to find pregnancy, parenting, and health information [32, 74, 100]. Lower income, single mothers may have greater online health information seeking behaviors because they may be more isolated and without family to provide support [74].

A well-designed digital intervention has the potential to be cost-effective compared to other delivery formats. The likelihood that our digital-based nutrition guidance system is effective at engaging this demographic and achieving behavioral adoption is high. Additionally, the bulk of the cost comes from the development of the digital system, which are nonrecurring, thereby leaving only minimal costs related to maintenance as the intervention is scaled up and new users are added [101].

### 3.8. Generalizability to Diverse Audiences

The target audience for this nutrition guidance system is first-time mothers in the United States. This system is intended to be applicable for a wide-range of income and educational levels as well as sociocultural groups and geographical locations. When reviewing the potential mediators of target behaviors, we included literature to capture a range of demographics, including specific subpopulations such as low income, WIC participants, and/or predominantly minority groups. The thorough review allowed us to identify those mediators which tended to be generalizable to all groups, and we focused our priority messaging around such mediators. Indeed, much of the research findings were generalizable to most groups. However, research findings that were too specific to a particular group were not included in final content. We maintained scientific accuracy of the content, by prefacing in the copy which population group was in the study. Finally, all photos on the website and emails were purposively selected to depict a diverse population.

Many studies target specific subpopulations of interest for a variety of factors including potential to benefit, research based on social welfare programs, and other existing relationships established with that population. Childhood obesity studies have often focused and continue to focus on subpopulations such as low income families [58, 102–106], particular racial/ethnic groups [102, 107–109], and children with overweight/obese mothers [59, 106, 110]. Healthy growth is important for all populations, and problems with overweight and obesity affect all race/ethnicities and socioeconomic groups. Scalability will require generalizability beyond previously studied subpopulations. New interventions will need to be developed with generalizability in mind and tested among the broader population such as our nutrition guidance system. However, cultural appropriateness will also be assessed using process measures as the intervention is rolled out and evaluated.

This multicomponent nutrition guidance system, based on its core characteristics, also has the capacity for dissemination to global contexts where undernutrition is prevalent. The rates of undernutrition remain high and the rates of obesity are rising in low and middle income countries (LMIC) [2]. The core messages targeting modifiable factors associated with obesity in essence support optimal growth, and many are comparable to the infant and young child feeding (IYCF) practices targeted for prevention of undernutrition. The rising obesity rates, potentially due to the nutrition transition and greater urbanization of LMIC, demand incorporation of physical activity, avoidance of sedentary behaviors, and adequate sleep into the global framework for achieving optimal child nutrition and development [2, 111]. To modify these behaviors, the identification of context specific mediators is the universal characteristic rather than any mediators themselves as context specific messages will likely enhance behavioral adoption more than general messages [112, 113]. Theory-based interventions are not common in global IYCF interventions but have shown success when used [114, 115]. Therefore this system will greatly contribute to the small evidence base of theory-based interventions in LMIC. Digital-based interventions are becoming more feasible with rising penetration of mobile and mobile broadband use in LMIC [116–118] and hold promise for greater coverage and cost effectiveness similar to other mobile health efforts [119–123]. Lastly, this system delivers education only, which means that there will be neither reliance on sustainable delivery of a product nor any violation of the International Code of Marketing of Breastmilk Substitutes. Ultimately, this multicomponent nutrition guidance system, due to this set of core characteristics, has a unique opportunity to address a majority of nutrition priorities found in LMIC and other global contexts.

### 3.9. Strengths and Limitations

The final set of characteristics for this multicomponent nutrition guidance system represents the strengths in the intervention design. First, the target behaviors are scientifically based on the most current understanding of potentially modifiable behaviors associated with childhood obesity and their underlying mediators. Second, the intervention has a theoretical foundation in the Social Cognitive Theory, Theory of Planned Behavior, Health Belief Theory, anticipatory guidance, and motivational interviewing. Third, the educational content and tools are delivered in a developmentally appropriate and sequential order. Fourth, the system is designed to engage a broad audience to ensure maximum applicability and scalability. Fifth, the delivery vehicles are digital which reflects the current communication and media trends and offers potential for greater cost effectiveness. Lastly, the system and its characteristics are designed to be generalizable for opportunities to disseminate to other global contexts.

The primary limitation to the design of this nutrition guidance system was the lack of formative research and pilot testing to guide the development of the intervention. The thorough review of the qualitative literature and existing formative research helps to mitigate this limitation as it captured many of the experiences of the target audience around the core messages to the point of saturation. As mentioned previously, prior to implementation in other populations, context specific mediators will need to be identified during additional formative research and pilot tested. Other related digital-based systems have been found acceptable and feasible in this population within Western cultures as well as Asian cultures [124–128]. Based on other formative and pilot research, mothers often appreciate personalized communication tailored to milestones [125, 127], not too frequent text messages with the ability to opt out [125, 127], email reminders [127], and content that is not alienating to mothers who are not breastfeeding [125]. Health care providers are reported to find digital systems acceptable as long as the content supports the recommendations they would give women and new mothers [124, 125]. As mentioned previously, our intervention content team and advisory board, who reviewed the entire intervention, included several health care providers, primarily Pediatricians, and Registered Dietitian Nutritionists. Much of the content was developed based on current recommendations from major health organizations such as the American Academy of Pediatrics. As the intervention was developed with evidence from other formative and pilot research and in line with current, standard pediatric practice, we anticipate that the digital-based system would be acceptable and feasible within our target population.

## 4. Conclusion

 A multicomponent nutrition guidance system designed and implemented as described constitutes a comprehensive intervention approach to address modifiable factors associated with early onset of childhood obesity and promote optimal growth. The rigorous method by which it was designed incorporates best practices for intervention, and this thorough description allows new users to adapt the system as necessary. The digital delivery format offers a cost-effective means to deliver the intervention in a manner that fits within the lifestyle of new mothers. Upon successful evaluation with an adequately designed clinical trial, this system can be scaled up to the proportions necessary to affect real and sustainable change in the goal to promote healthy nutrition and growth in the first 1000 days.

## Figures and Tables

**Table 1:** Alignment of theoretical constructs with simplified mediators in research content tables.

*Motivational constructs*	

Knowledge	(i) Behavioral beliefs^1^, outcome expectations^2^, perceived benefits^3^
	(ii) Perceived risk (susceptibility + severity)^2^

Facilitators	(i) Existing positive or strong
	(a) Behavioral beliefs^1^, outcome expectations^2^, perceived benefits^3^
	(b) Outcome evaluations^1^, attitudes^1^, outcome expectancies^2^
	(c) Perceived risk^3^
	(d) Social norms^1^, social outcome expectations^2^
	(e) Self-efficacy^2,3^, perceived behavioral control^1^

Barriers	(i) Existing positive or strong
	(a) Behavioral beliefs^1^, outcome expectations^2^, perceived benefits^3^
	(b) Outcome evaluations^1^, attitudes^1^, outcome expectancies^2^
	(c) Perceived risk^3^
	(d) Social norms^1^, social outcome expectancies^2^
	(e) Self-efficacy^2,3^, perceived behavioral control^1^
	(ii) Environmental constraints^2,3^

*Capacity to act constructs*	

Knowledge	(i) Behavioral capability (relevant background knowledge)^1^

Barriers	(i) Increasing self-efficacy to overcome existing barriers^2^
	(ii) Self-regulation^2^

Instruction	(i) Behavioral capability^2^
	(ii) Increase self-efficacy^2^

^1^Theory of Planned Behavior.

^2^Social Cognitive Theory.

^3^Health Belief Model.

**Table 2:** Examples of intervention features for behavioral adoption.

Theoretical constructs	Theoretical techniques	Intervention examples
Behavioral beliefs, social norms, perceived risk	(1) Provide information on consequences (2) Provide normative information about others' behaviors	(i) Article on benefits of breastfeeding (ii) Articles mentioning recommendations from AAP and other groups (iii) Article describing statistics about breastfeeding (iv) Slideshow with picture of average parents role modeling healthy eating

Outcome evaluations, attitudes	(1) Acknowledgement of feelings, motivations (2) Emotion-based messages	(i) “I promise” statement to help mothers articulate their commitment to their baby and why

Self-efficacy and improving self-efficacy via social modeling, mastery experiences, social persuasion	(1) Barrier identification (2) Model/demonstrate the behavior (3) Provide general encouragement (4) Prompt goal setting	(i) Article on common challenges during breastfeeding (ii) Video of breastfeeding mother (iii) Encouraging text messages and statements within articles (iv) Goal setting tool

Relevant knowledge	(1) Feeding and nutrition knowledge	(i) Article on how breastmilk is produced (ii) Glossary to define terms

Behavioral capability	(1) Provide instruction (2) Demonstrate the behavior	(i) Video of woman demonstrating how to latch a baby onto her breast (ii) Stills of how to swaddle a baby (iii) Table showing suggested bottle volumes by age (iv) Step-by-step instructions for introducing solid foods

Self-regulation	(1) Prompt goal setting (2) Prompt self-monitoring of behavior (3) Stress management	(i) Growth tracker (ii) Goal setting tool (iii) Baby/family simplified meal planner (iv) Call to action where mothers are prompted to upload photo of their child doing one of the core messages (v) Breastfeeding tracker (vi) Taste tracker (vii) Article on taking care of oneself after delivery (viii) Articles reminding mothers to reach out to social support system (ix) Tone that recognizes all emotions are normal

**Table 3:** Core messages and timing of delivery to provide anticipatory guidance.

Core messages	Third trimester	Birth	2 mo	4 mo	6 mo	8 mo	10 mo	12 mo	14 mo	16 mo	18 mo	20 mo	22 mo
Provide breastmilk	**✓**	**✓**	**✓**	**✓**	**✓**	**✓**	**✓**						

Utilize responsive feeding practices	**✓**	**✓**	**✓**	**✓**	**✓**	**✓**	**✓**	**✓**	**✓**	**✓**	**✓**	**✓**	**✓**

Provide nutritious complementary foods and beverages at the appropriate developmental stage			**✓**	**✓**	**✓**	**✓**	**✓**	**✓**	**✓**	**✓**	**✓**	**✓**	**✓**

Exclude sugar sweetened beverages for infants and limit for toddlers				**✓**	**✓**	**✓**	**✓**	**✓**	**✓**	**✓**	**✓**	**✓**	**✓**

Foster healthy eating behaviors through shared family meals and mealtime routines					**✓**	**✓**	**✓**	**✓**	**✓**	**✓**	**✓**	**✓**	**✓**

Limit TV and screen viewing time				**✓**	**✓**	**✓**	**✓**	**✓**	**✓**	**✓**	**✓**	**✓**	**✓**

Provide opportunities for physical activity		**✓**	**✓**	**✓**	**✓**	**✓**	**✓**	**✓**	**✓**	**✓**	**✓**	**✓**	**✓**

Ensure that the infant and toddler have adequate sleep	**✓**	**✓**	**✓**	**✓**	**✓**	**✓**	**✓**	**✓**	**✓**	**✓**	**✓**	**✓**	**✓**
